# Preparation of High-Performance Activated Carbon from Coffee Grounds after Extraction of Bio-Oil

**DOI:** 10.3390/molecules26020257

**Published:** 2021-01-06

**Authors:** Jie Ren, Nanwei Chen, Li Wan, Guojian Li, Tao Chen, Fan Yang, Shuiyu Sun

**Affiliations:** 1 Guangdong Polytechnic of Environmental Protection Engineering, Foshan 528216, China; renjie18211489691@163.com (J.R.); cnw346026246@163.com (N.C.); wanli18814140916@126.com (L.W.); gjli0223@163.com (G.L.); fenix1985@126.com (F.Y.); 2 School of Environment, South China Normal University, University Town, Guangzhou 510006, China; tao.chen@m.scnu.edu.cn; 3 School of Environmental Science and Engineering, Guangdong University of Technology, Guangzhou 510006, China

**Keywords:** coffee grounds, bio-oil, phosphoric acid, activated carbons, adsorption

## Abstract

In this study, a new method for economical utilization of coffee grounds was developed and tested. The resulting materials were characterized by proximate and elemental analyses, thermogravimetric analysis (TGA), Fourier transform infrared spectroscopy (FTIR), scanning electron microscopy (SEM), and N_2_ adsorption–desorption at 77 K. The experimental data show bio-oil yields reaching 42.3%. The optimal activated carbon was obtained under vacuum pyrolysis self-activation at an operating temperature of 450 °C, an activation temperature of 600 °C, an activation time of 30 min, and an impregnation ratio with phosphoric acid of 150 wt.%. Under these conditions, the yield of activated carbon reached 27.4% with a BET surface area of 1420 m^2^·g^−1^, an average pore size of 2.1 nm, a total pore volume of 0.747 cm^3^·g^−1^, and a t-Plot micropore volume of 0.428 cm^3^·g^−1^. In addition, the surface of activated carbon looked relatively rough, containing mesopores and micropores with large amounts of corrosion pits.

## 1. Introduction

It is well-known that climate change is mainly caused by greenhouse gas emissions from natural systems and human activities. So far, human activities have caused about 1.0 °C of global warming above the pre-industrial level, and this is likely to reach 1.5 °C between 2030 and 2052 if the current emission rates persist [1]. In 2015, the Paris Agreement was introduced with the main objective of limiting the global temperature increase to 2 °C by 2100 and pursuing efforts to limit the increase to 1.5 °C, which implies significant changes in the global energy supply system [2,3]. To reach the milestone launched by the European Commission for the use of energy sources, by 2030, at least 20% of overall energy consumption should be satisfied with the use of renewable energy sources and, also, at least 10% of transportation fuel demand should be fulfilled with biofuels [4]. The biofuels can be referred to as the gaseous and liquid fuels for the transport sector that are generally produced from biomass [5]. The use of biomass in liquid biofuels production has attracted increasing interest worldwide due to its abundance, renewability, and low cost [6].

Coffee, with the spent coffee ground as waste, is known as one of the most-consumed beverages worldwide [7]. Between 2016 and 2019, world coffee consumption increased by 4.5% and reached 9.92 million tons [8]. Coffee consumption is projected to reach 12.24 million tons by 2030 [9]. Coffee grounds contain about 47.3% oil [10], mainly composed of 80~90 wt.% glycerides, like free fatty acids [11]. The high content of organic matter and low content of ash in coffee grounds make them potential biomass [12,13]. However, most coffee grounds end up disposed of in landfills, causing environmental and ecological problems [14]. Coffee grounds demand high oxygen during the decay process and release residual caffeine, tannin, and polyphenol into the environment [15]. Therefore, the development of new methods for the economical utilization of coffee grounds is highly desirable to produce energy and reduce the negative impact on the environment.

**Figure 1:**
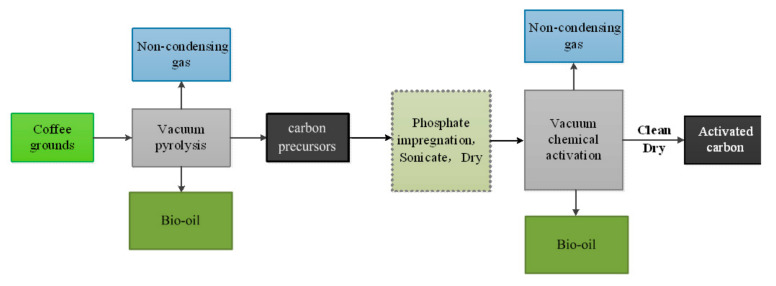
Refining scheme.

In this study, we aim to develop a novel economical route for utilization of coffee grounds (Figure 1). The currently used methods for manufacturing of activated carbon are mainly based on physical and chemical activations [16,17]. To the best of the authors’ knowledge, there is no study on producing activated carbon via the two-stage activation (pyrolyzation + chemical activation) method from coffee grounds. The two-stage activation method can improve the added value of coffee grounds, and the specific surface areas (BET) of the activated carbon can be favorably compared with other activated carbon produced from coffee grounds [18,19]. During the pyrolysis self-activation of coffee grounds, the self-activation temperature of vacuum pyrolysis was first determined by thermogravimetry. Both the bio-oil and carbon precursors were then obtained by vacuum pyrolysis of coffee grounds at the optimized activation temperature. Finally, activated carbon was prepared from carbon precursors impregnated by phosphoric acid aqueous solution under different activation conditions. The effects of the impregnation ratio with phosphoric acid, activation temperature, and activation time on the structure and morphology of activated carbon were investigated by various analytical methods.

## 2. Materials and Methods

### 2.1. Experimental Setup

**Figure 2:**
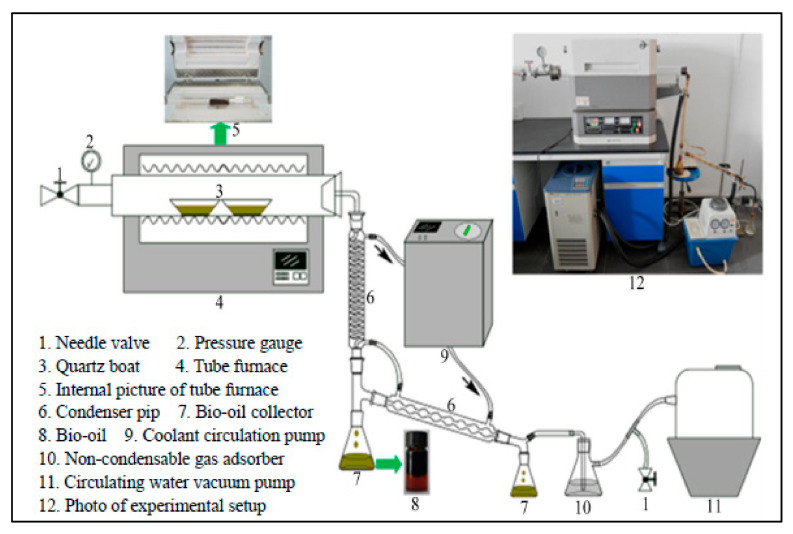
Schematic diagram of two-stage activation.

The employed experimental apparatus is provided in Figure 2, mainly composed of a programmed furnace, a condensation collecting system, a non-condensable gas absorption system, and a vacuum chamber. The programmed furnace (SKG06123K, Tianjin, China) possessed a rated power of 2.5 kW, controllable temperatures ranging from ambient temperature to 1200 °C, and a heating rate from 10 to 40 °C·min^−1^. The length, inside, and outside diameters of the quartz tube were 1 m, 55 nm, and 60 mm, respectively. The condensation collecting system was composed of at least two condenser tubes, two conical flasks, and a coolant circulation pump (DLSB-5/20, Zhengzhou, China). The temperature of the condensation collecting system was maintained at 10 °C using a coolant circulation pump. The employed cooling medium was water, and the gas absorption system was composed of a conical flask and a strong oxidizing solution. The vacuum chamber contained quartz tube, a vacuum pump, and an air pressure valve. The vacuum chamber was equipped with a controllable vacuum from 10 kPa to 101.325 kPa, and alkaline water was used in the vacuum pump.

### 2.2. Preparation of Optimal Activated Carbon

#### 2.2.1. Vacuum Pyrolysis Self-Activation

The coffee grounds used here were collected from an instant coffee factory in the city of Dongguan, China. The collected coffee grounds were first washed with distilled water to eliminate the impurities (dust and water-soluble substances), and dried at 105 °C for 48 h.

The dried coffee grounds were then subjected to vacuum pyrolysis self-activation. To this end, a 40 g dried coffee grounds sample was placed in the programmed furnace with quartz tube under vacuum conditions (10 kPa). Next, the material was heated to 450 °C at a 10 °C·min^−1^ heating rate, then held at this temperature for 1 h. After cooling down to ambient temperature, the carbon precursor and bio-oil were obtained.

#### 2.2.2. Chemical Activation

To test the effects of activation temperature, 10 g of dried carbon precursor was impregnated with 50 g of phosphoric acid aqueous solution (30 wt.%) for 2 h to form slurries. The suspensions were sonicated for 1 h in an ultrasound bath (40 kHz) then dried at 105 °C for 24 h. Next, the specimens were heated to different temperatures (400, 500, 600, and 700 °C) under vacuum conditions (20 kPa) for 30 min at a 10 °C·min^−1^ heating rate.

To evaluate the influence of the impregnation ratio with phosphoric acid (Xp: phosphoric acid/dry carbon precursor, *g*/*g*, wt.%), 10 g of dried carbon precursor was impregnated for 2 h with different quantities (16.67, 33.33, 50, and 66.67 g) of phosphoric acid aqueous solutions (30 wt.%) to form slurries. The suspensions were then sonicated for 1 h in an ultrasound bath (40 kHz) and dried at 105 °C for 24 h. Next, the obtained specimens were heated to 600 °C under vacuum conditions (20 kPa) for 30 min at a 10 °C·min^−1^ heating rate.

To examine the effect of activation time, 10 g of dried carbon precursor was impregnated for 2 h with 50 g of phosphoric acid aqueous solution (30 wt.%) to form slurries. The suspension was then sonicated for 1 h in an ultrasound bath (40 kHz) and dried at 105 °C for 24 h. Next, it was heated to 600 °C under vacuum conditions (20 kPa) at 10 °C·min^−1^ for different activation times (20, 30, 40, and 50 min). After cooling to ambient temperature in the vacuum chamber, the obtained specimens were subjected to thorough washing with hot water until pH neutrality was reached, then dried at 105 °C for at least 24 h. The final materials were then ground and sieved to yield particles with diameters < 0.074 nm, then kept in closed containers for future experiments.

### 2.3. Yields of Carbon Precursor, Bio-Oil, Non-Condensable Gas, and Activated Carbon

The yields of carbon precursor, bio-oil, non-condensable gas, and activated carbon were estimated by means of Equations (1)–(4):

(1) $$Y_{cp}=\frac{m_{cp}}{m_{0}}\times 100\%$$

(2) $$Y_{L}=\frac{m_{L}}{m_{0}}\times 100\%$$

(3) $$Y_{G}=\frac{m_{0}-m_{cp}-m_{L}}{m_{0}}\times 100\%$$

(4) $$Y_{ac}=\frac{m_{ac}}{m_{0}}\times 100\%$$

where $Y_{cp}$, $Y_{L}$, $Y_{G}$ and $Y_{ac}$ are the yields (wt.%) of carbon precursor, bio-oil, non-condensable gas and activated carbon, respectively. $m_{0}$, $m_{cp}$, $m_{L}$ and $m_{ac}$ represent the weight (g) of dried coffee grounds, carbon precursor, bio-oil and activated carbon, respectively.

### 2.4. Characterization

#### 2.4.1. Thermogravimetry, Structural, and Morphological Analyses

The thermogravimetry analysis (TGA) was carried out under an N_2_ (30 mL·min^−1^) atmosphere using a NETZSCH STA409PC simultaneous analyzer (STA409PC, NETZSCH, Selb, Germany). The TGA tests were performed at a heating rate of 10 °C·min^−1^ from ambient temperature to 800 °C. About 9.5–10.5 mg specimens were loaded into the TGA unit for analysis.

The Fourier transform infrared (FT-IR) spectra were collected on a Thermo Fisher FTIR spectrometer (Nicolet 380, Thermo Fisher Scientific, Gloucester, UK). The mixtures containing specimen and KBr (1:100) were first ground in an agate mortar and then mixed uniformly and compressed using a hydraulic press machine. The FTIR spectra were recorded from 400 to 4000 cm^−1^.

The morphologies of the specimens were viewed by scanning electron microscopy (SEM) (S-3400N, Hitachi, Japan).

#### 2.4.2. Proximate and Elemental Analyses

The proximate analyses were carried out according to GB/T 28731-2012 (National Standard, China). For moisture analysis, coffee grounds (1 ± 0.1 g) were dried at 105 ± 2 °C in air to obtain constant mass. For determination of ash content, coffee grounds (1 ± 0.1 g) were heated to constant mass in a muffle furnace, at a heating rate of 5 °C·min^−1^ up to 550 ± 10 °C. To estimate volatile content, coffee grounds (1 ± 0.1 g) were heated at 900 ± 10 °C in a muffle furnace for 7 min. The fixed carbon was calculated by means of Equation (5):

(5) FC=100%−V−A−M

where FC, V, A, and M are the contents (wt.%) of fixed carbon, volatile ash, and moisture, respectively.

The C, H, N, S, and P contents (mass %) of coffee grounds, carbon precursor, and activated carbons were measured by elemental analysis (Perkin Elmer Series II 2400, Waltham, MA, USA). The O content was calculated according to Equation (6):

(6) O=100%−C−H−N−S−A−M

where O, C, H, N, S, A, and M are the contents (wt.%) of O, C, H, N, S, ash, and moisture, respectively.

#### 2.4.3. N_2_ Adsorption–Desorption Profiles at 77 K

The N_2_ adsorption–desorption isotherms of the carbon precursor and activated carbons were measured on an automatic adsorption instrument (ASAP 2020M, Micromeritics, Norcross, GA, USA) at 77 K [20]. The specific surface areas of the specimens were calculated by the BET equation, assuming the nitrogen molecule area of 0.162 nm^2^. The total pore volume was estimated as liquid volume of the adsorbate adsorbed at P/Po = 0.99 [21]. The average pore sizes were estimated by 4 V/A, where V is total pore volume and A is BET surface area. The micropore volume was determined by means of the t-Plot method. The pore size distributions were obtained by the BJH method [22].

The N_2_ adsorption–desorption isotherms of the carbon precursor and activated carbons were measured on an automatic adsorption instrument (ASAP 2020M, Micromeritics, Norcross, GA, USA) at 77 K [20]. The specific surface areas of the specimens were calculated by the BET equation, assuming the nitrogen molecule area of 0.162 nm^2^. The total pore volume was estimated as liquid volume of the adsorbate adsorbed at P/Po = 0.99 [21]. The average pore sizes were estimated by 4 V/A, where V is total pore volume and A is BET surface area. The micropore volume was determined by means of the t-Plot method. The pore size distributions were obtained by the BJH method [22].

#### 2.4.4. Bio-Oil Composition Analysis

The bio-oil phase was analyzed following a previously reported method [23]. Briefly, predilution of the bio-oil (no moisture) was first performed in acetone then passed through the organic filter membrane (aperture size 0.22 µm) using an injector. Next, 0.2 µL sample solution was analyzed using gas chromatography/mass spectrometry (GC/MS) (7890A/5975C, Agilent, Santa Clara, CA, USA) at 250 °C, a split ratio of 10:1, and using helium as the carrier gas at a flow rate of 20 mL·min^−1^. An AHP-5MS GC column (30 m × 250 µm × 0.25 µm) was used to separate the components, and heated from 100 °C to 250 °C at 10 °C·min^−1^ for 5 min.

## 3. Results and Discussion

### 3.1. Thermal Properties of Coffee Grounds

**Figure 3:**
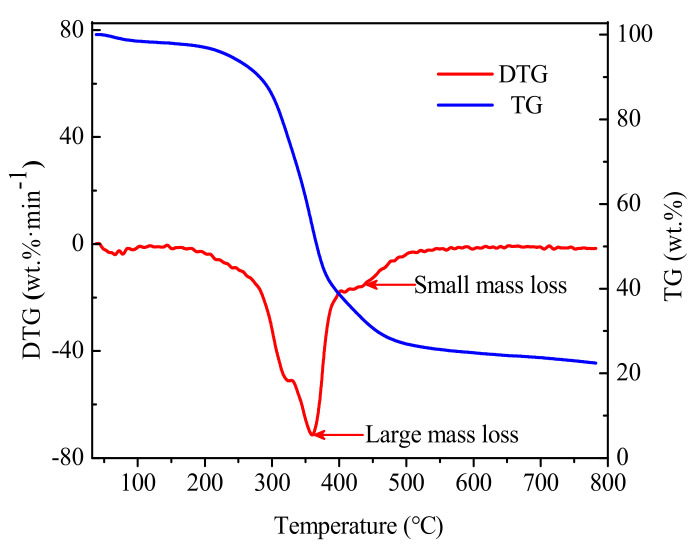
Thermogravimetric graph from coffee grounds under the heating rate of 10 °C·min^−1^ and 30 mL·min^−1^ nitrogen flow.

Figure 3 shows the TG and derivative thermogravimetry (DTG) curves of coffee grounds under N_2_ (30 mL·min^−1^) at a heating rate of 10 °C·min^−1^ from ambient temperature to 800 °C. The mass loss of coffee grounds showed three stages. Below 150 °C, the mass loss was estimated to be 2.68% and attributed to loss of moisture from coffee grounds. In the range of 150 °C < T < 500 °C, the mass loss was calculated as 66.04% and related to evaporation of main volatile matters, tars elimination, and carbonization (primary and double-time carbonizations). In the third stage (500 °C < T < 800 °C), the coffee grounds were almost totally carbonized with a remaining mass of only 22.48%. The DTG profiles in the second step (150 °C < T < 500 °C) showed large and small mass losses. The large weight loss would correspond to elimination of volatile matters and tars, as well as complete primary and double-time carbonization. The second small mass loss observed at 450 °C could be associated with decomposition of oxygenated surface groups [18]. Therefore, the optimum vacuum pyrolysis self-activation temperature for high yield of bio-oil and microporous carbon precursors was selected as 450 °C.

### 3.2. Proximate and Elemental Analyses

**Figure 4:**
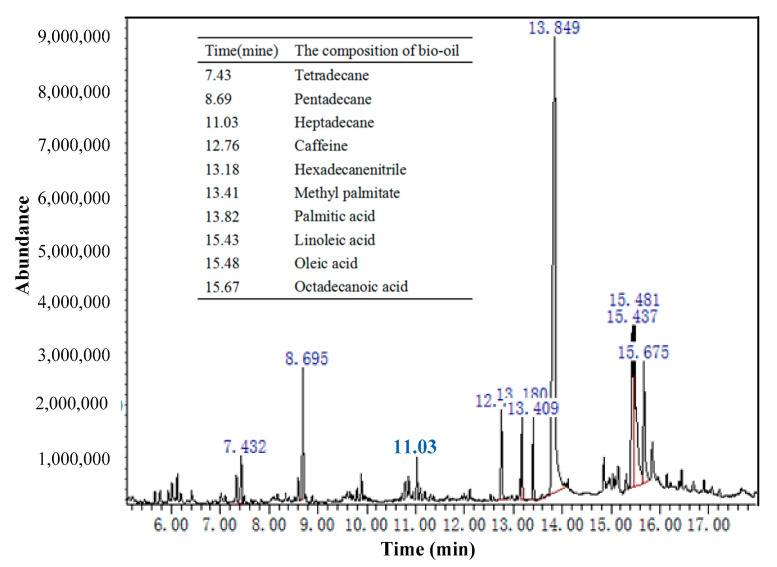
GC/MS chromatograms of bio-oil.

Table 1 lists the results of proximate and elemental analyses of coffee grounds and carbon precursors. The high content of volatiles (74.82%) in the coffee grounds demonstrated their relevance for the production of bio-oil. The content of volatiles (8.79%) in the carbon precursor was lower than in coffee grounds, indicating conversion of some volatiles into bio-oil in coffee grounds during vacuum pyrolysis at 450 °C. The bio-oil was collected by the condensation collecting system, and the yields of bio-oil, carbon precursors, and non-condensable gas were estimated to be 42.3%, 31.4%, and 26.3%, respectively. The bio-oil was analyzed by GC/MS (Figure 4), and determined to contain tetradecane, pentadecane, heptadecane, caffeine, hexadecanenitrile, methyl palmitate, palmitic acid, linoleic acid, oleic acid, and octadecanoic acid. Meanwhile, the carbon precursors containing high carbon content (86.85%) and low ash (2.05%) might be used as raw material in production of activated carbon [24].

### 3.3. Effect of Activation Conditions on Various Factors

#### 3.3.1. Yield

Table 2, Table 3 and Table 4 gather the effect of different activation conditions on yield of activated carbon. The increase in activation temperature, activation time, and the impregnation ratio from 400 °C, 20 min, and 50 wt.% to 700 °C, 50 min, and 200 wt.% reduced the yield of activated carbon from 29.9%, 28.1%, and 28.5% to 24.6%, 26.3%, and 26.6%, respectively. The yield of activated carbon showed a common trend at different activation conditions, decreasing as the activation temperature, activation time, and impregnation ratio increased. This was associated with the chemical activation with H_3_PO_4_ impregnation, in which the increase in activation temperature, activation time, and the impregnation ratio led to the release of more volatile products due to intensified dehydration and elimination reactions [18].

#### 3.3.2. Activation Temperature on BET Surface Area

Table 2 lists the activation conditions and effect of activation temperature on BET surface area of activated carbon. As the activation temperature rose from 400 to 600 °C, the BET surface area of activated carbon increased from 102 m^2^·g^−1^ to 1420 m^2^·g^−1^. This is related to the formation of pores due to increased devolatilization and a carbon–H_2_O reaction. At 700 °C, the BET surface area of activated carbon was recorded as 962 m^2^·g^−1^. A further increase in the activation temperature to 700 °C reduced the BET surface, mainly due to the excessive burn-off of carbon constituents at high temperatures. In addition, H_3_PO_4_ formed a linkage layer composed of phosphate and polyphosphate esters, leading to a decrease in BET surface area.

#### 3.3.3. Activation Time on BET Surface Area

Table 3 displays the activation conditions and the influence of the activation time on the BET surface area of activated carbon. The increase in activation time from 20 to 30 min enhanced the BET surface area of activated carbon from 923 m^2^·g^−1^ to 1420 m^2^·g^−1^. However, from 30 to 50 min, the BET surface area of activated carbon decreased from 1420 m^2^·g^−1^ to 873 m^2^·g^−1^, which is attributed to intense apparent pore structure development during the initial activation time. Therefore, the BET surface areas reached maxima at 30 min. The increase in activation time from 30 to 50 min reduced the BET surface area because of enlargement in the pore structure after exposure to a continuous high temperature.

#### 3.3.4. Impregnation Ratio on BET Surface Area

Table 4 provides the activation conditions and influence of the impregnation ratio on the BET surface area. The enhancement in the impregnation ratio from 50 to 150 wt.% increased the BET surface area of activated carbon from 628 m^2^·g^−1^ to 1420 m^2^·g^−1^. From 150 to 200 wt.%, the BET surface area of activated carbon decreased from 1420 m^2^·g^−1^ to 994 m^2^·g^−1^. In other words, the BET surface area rose to a maximum and then decreased with a further increase in the impregnation ratio, consistent with previous reports [25]. The addition of more acid was not beneficial as it did not induce further action, and probably formed an insulating layer (or skin) covering the particles. This, in turn, inhibited the activation process and contact with the surrounding atmosphere [26].

### 3.4. Properties of Optimal Activated Carbon

#### 3.4.1. Morphology

**Figure 5:**
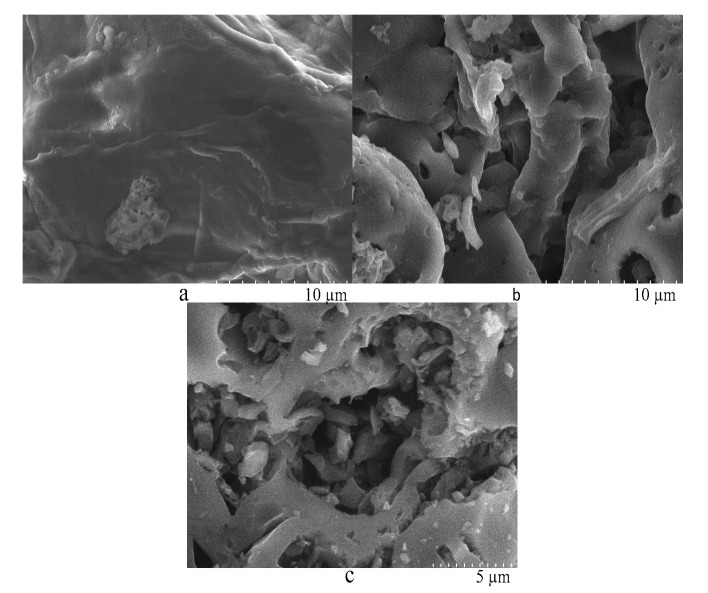
The SEM images of (**a**) coffee grounds, (**b**) carbon precursor, and (**c**) activated carbons.

The SEM images of coffee grounds (CGs), carbon precursors (CPs), and activated carbons (ACs) are shown in Figure 5. The surface structure of CG looked compact and nonporous (Figure 5a), unfavorable for phosphoric acid penetration. Here, CP was issued from the CG vacuum pyrolysis used to collect bio-oil and open a transmission channel for phosphoric acid. In Figure 5b, the CP surface exhibits a certain number of micropores, beneficial to phosphoric acid penetration. The wide opening of transmission channels for phosphoric acid induced its facile penetration, improving the activation efficiency of phosphate ions. Both activations of vacuum pyrolysis and phosphoric acid promoted the transformation of CG into AC. In Figure 5c, the AC surface looks relatively rough with large amounts of corrosion pits.

#### 3.4.2. Structure

**Figure 6:**
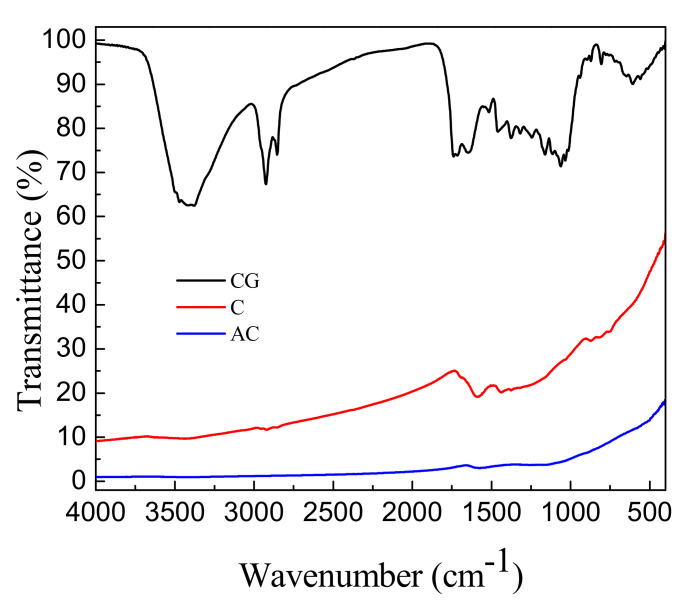
FTIR spectra of coffee grounds (CG), carbon precursors (CP), and activated carbons (AC).

The FTIR spectra of coffee grounds (CGs), carbon precursor (CP), and activated carbon (AC) are shown in Figure 6. The peak at 3400 cm^−1^ obviously appeared in the absorption profile of CG, ascribed to O–H stretching vibration [27] that issued mainly from long-chain carboxylic acids. The absorption peaks at 2700–3000 cm^−1^ were ascribed to C–H vibrations, indicating the presence of alkyl groups in CG [10]. The complex absorption peaks between 1000 cm^−1^ and 1700 cm^−1^ were related to C–H bending of cellulose and hemicelluloses [28], as well as C=O stretching from carboxylic acids and ester groups [10,29]. In particular, the broad band between 600 cm^−1^ and 1000 cm^−1^ was ascribed to C–H (Ar) bending [30,31], corresponding to aromatic ring compounds. During preparation of activated carbon, the absorption peaks at 1000–1750 cm^−1^, 2700–3000 cm^−1^, and 3400 cm^−1^ reduced significantly or even vanished, meaning breaking of the functional groups of C=O, C–H, and O–H.

#### 3.4.3. Pore Size

**Figure 7:**
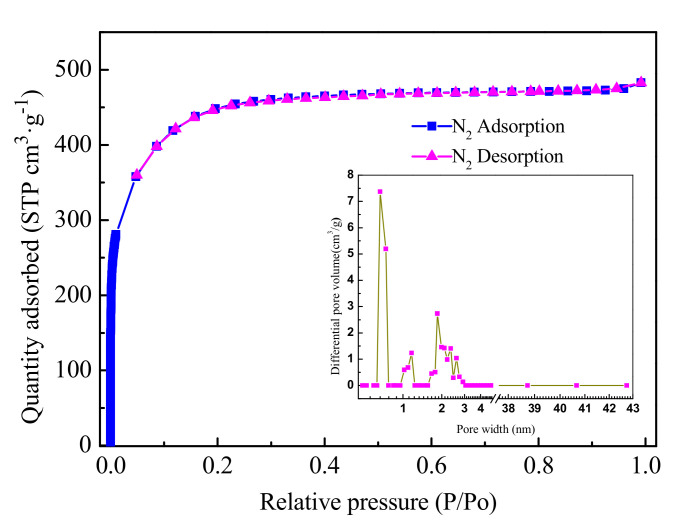
N_2_ adsorption–desorption isotherms at 77 K and differential pore size of the optimum activated carbons.

Figure 7 depicts the N_2_ adsorption–desorption isotherms and differential pore size of activated carbons prepared under the optimum conditions of vacuum pyrolysis self-activation at an operating temperature of 450 °C, an activation temperature of 600 °C, an activation time of 30 min, and an impregnation ratio of 150 wt.%. The BET surface area of activated carbons was estimated to be 1420 m^2^·g^−1^, with an average pore size of 2.1 nm, a total pore volume of 0.747 cm^3^·g^−1^, and a t-Plot micropore volume of 0.428 cm^3^·g^−1^. The N_2_ adsorption isotherm of activated carbon exhibited a Type I hysteresis with an almost horizontal plateau at higher relative pressures, indicating highly microporous materials with a narrow pore size distribution. The desorption branch revealed a small hysteresis loop of type H4, suggesting elevated microporosity with slit-like pores [32,33,34]. The activated carbon was obviously microporous. The differential pore size distribution illustrated that activated carbons mainly exhibited a three-peak curve at 0.67, 1.17, and 1.84 nm, with pore diameter below 2.91 nm. Hence, the pore size distribution of prepared activated carbons was narrow.

## 4. Conclusions

A novel method for economical utilization of coffee grounds was developed for production of bio-oil and activated carbon. Vacuum pyrolysis self-activation and chemical activation with H_3_PO_4_ were found to be important steps in carbon activation. Yields reaching 42.3% of bio-oil and 31.4% of carbon precursors were obtained by vacuum pyrolysis self-activation of coffee grounds. The carbon precursor surface exhibited a certain number of micropores, beneficial to phosphoric acid penetration. The wide opening in transmission channels of phosphoric acid induced its facile penetration, hence improving the activation efficiency of phosphate ions. Under the optimal chemical activation conditions, in amounts reaching 27.4%, coffee grounds were transformed into high-performance activated carbon with a BET surface area of 1420 m^2^·g^−1^, a total pore volume of 0.747 cm^3^·g^−1^, and a t-Plot micropore volume of 0.428 cm^3^·g^−1^. The optimal activated carbon surface looked relatively rough with large amounts of corrosion pits and a narrow pore size. Our approach opens doors for the preparation of activated carbon from coffee grounds by the two-stage activation method, which could be of great interest for much biomass. It is anticipated that this method will increase the economic value of biomass.

## Figures and Tables

**Table 1:** Proximate analysis and elemental analysis of the samples.

Samples	Proximate Analysis				Elemental Analysis				
	M	V	A	FC	C	H	O	N	S
Coffee Grounds	2.69%	74.82%	0.56%	21.93%	56.94%	15.23%	20.88%	2.76%	0.98%
Carbon Precursors	2.31%	8.79%	2.05%	86.85%	72.08%	8.77%	9.40%	4.75%	0.64%

**Table 2:** Activation conditions, yield, and BET surface area of activated carbon.

Activation Conditions		Yield (%)	BET Surface Area S_BET_ (m^2^·g^−1^)
Other Conditions	T (°C)		
T = 30 min	400	29.9	102
X_p_ = 150 wt.%	500	28.3	651
Pressure: 20 kPa	600	27.4	1420
Heating Rate: 10 °C·min^−1^	700	24.6	962

**Table 3:** Activation conditions, yield, and BET surface area of activated carbon.

Activation Conditions		Yield (%)	BET Surface Area S_BET_ (m^2^·g^−1^)
Other Conditions	t (min)		
T = 600 °C	20	28.1	923
X_p_ = 150 wt.%	30	27.4	1420
Pressure: 20 kPa	40	26.7	1080
Heating Rate: 10 °C·min^−1^	50	26.3	873

**Table 4:** Activation conditions, yield, and BET surface area of activated carbon.

Activation Conditions		Yield (%)	BET Surface Area S_BET_ (m^2^·g^−1^)
Other Conditions	Xp (wt.%)		
T = 600 °C	50	28.5	628
T = 30 min	100	27.7	887
Pressure: 20 kPa	150	27.4	1420
Heating Rate: 10 °C·min^−1^	200	26.6	994

## Data Availability

Data of present study, will be available on request from corresponding author.
